# The role of ultralarge multimers in recombinant human von Willebrand factor – a review of physico-and biochemical studies and findings in *in vivo* models and in humans with von Willebrand disease

**DOI:** 10.5482/HAMO-17-04-0018

**Published:** 2017-12

**Authors:** Peter L Turecek, Michael Spannagl, Thorsten Kragh, Guenter Allmaier, Marietta Turecek, Gerald Schrenk, Herbert Gritsch, Ortrun Obermann-Slupetzky, Bruce M Ewenstein, Leonard A Valentino

**Affiliations:** 1 Baxalta Innovations GmbH, Vienna, Austria, now part of Shire; 2 Department of Transfusion Medicine and Hemostasis, University Hospital Munich, Munich, Germany; 3 Institute of Chemical Technologies and Analytics, Vienna University of Technology, Vienna, Austria; 4 Shire, Cambridge, MA, USA; 5 Shire and Rush University, Chicago, IL, USA

**Keywords:** Recombinant von Willebrand Factor, von Willebrand Disease, multimers, hemostasis, Rekombinanter von Willebrand Faktor, von Willebrand Syndrom, Multimere, Hämostase

## Abstract

Ultralarge multimers (ULM) of VWF are considered to be the most active with respect to binding to platelets and to subendothelial structures and therefore are of critical importance for the function of VWF in stabilizing the primary hemostatic plug. In contrast to plasma-derived FVIII-VWF concentrates, human rVWF obtained from mammalian cell culture retains the full-spectrum of intact multimers, including ULM, as physiologically formed in the Golgi apparatus and stored in platelet α-granules and endothelial cell Weibel–Palade bodies. In the course of physico and biochemical, functional and animal studies, rVWF exhibited superiority in structure and function compared to pdVWF. These effects seemed to correlate with the multimer size and therefore might be attributed to the presence of ULM in rVWF preparations. The pharmacokinetic (PK), safety and efficacy characteristics seen in preclinical studies were further demonstrated in clinical trials.

## Zusammenfassung

Den hochmolekularen Multimeren des VWF wird die höchste Aktivität zugeschrieben, an Thrombozyten und subendotheliale Strukturen zu binden und sind daher von kritischer Relevanz für die hämostatischen Funktionen von VWF bei der Stabilisierung des Thrombus. Im Gegensatz zu allen FVIII/VWF-haltigen Konzentraten, die aus Plasma gewonnen werden, behält humaner rVWF, der aus Säugetierzellkulturen gewonnen wird, ein intaktes Multimerspektrum, welches physiologisch im Golgi Apparat gebildet und in den α-Granula der Thrombozyten und in Weibel-Palade-Körperchen der Endothelzellen gespeichert wird. Im Rahmen physiko-und biochemischer, funktioneller und tierexperimenteller Studien wurde festgestellt, dass rVWF bessere Eigenschaften in Struktur und Funktion als plasmatischer VWF besitzt. Diese Eigenschaften korrelieren möglicherweise mit der Größe der Multimere und könnten daher durch die hochmolekularen Multimere in rVWF hervorgerufen werden. In den klinischen Studien, die mit rVWF im Vergleich zu einem plasmatischen FVIII-VWF-Konzentrat durchgeführt wurden, konnten die für rVWF anderen pharmakologischen und pharmakokinetischen Eigenschaften als für pdVWF bestätigt werden.

Ultralarge multimers (ULM) of VWF are considered to be the most active with respect to binding to platelets and to subendothelial structures and therefore are of critical importance for the function of VWF in stabilizing the primary hemostatic plug. In contrast to plasma-derived FVIII-VWF concentrates, human rVWF obtained from mammalian cell culture retains the full-spectrum of intact multimers, including ULM, as physiologically formed in the Golgi apparatus and stored in platelet α-granules and endothelial cell Weibel–Palade bodies. In the course of physico and biochemical, functional and animal studies, rVWF exhibited superiority in structure and function compared to pdVWF. These effects seemed to correlate with the multimer size and therefore might be attributed to the presence of ULM in rVWF preparations. The pharmacokinetic (PK), safety and efficacy characteristics seen in preclinical studies were further demonstrated in clinical trials.

**Korrespondenzadresse**
Dr. Peter L Turecek
Shire
Industriestrasse 67
Vienna, Austria
Email: peter.turecek@shire.com

**Zur Funktion der großen Multimere in rekombinantem humanem von Willebrand Faktor – Ein Review physiko-und biochemischer Studien und von Ergebnissen aus Tiermodellen und klinischen Studien in Patienten mit von Willebrand Syndrom**

**Haemostaseologie 2017; 37 (Suppl 1): S15–S25**
**https://doi.org/10.5482/HAMO-17-04-0018**
received: April 11, 2017 accepted: October 9, 2017
© 2017. The Author(s).
This is an open access article published by Thieme under the terms of the Creative Commons Attribution License, permitting unrestricted use, distribution, and reproduction so long as the original work is properly cited.
(https://creativecommons.org/licenses/by/4.0/).
Georg Thieme Verlag KG, Oswald-Hesse-Straße 50, 70469 Stuttgart, Germany

## Introduction

Von Willebrand factor (VWF) is the largest known glycoprotein circulating in human blood plasma and is composed of a series of multimers with molecular weights ranging from 600 to 20,000 kDa. VWF has three major functions in hemostasis, 1) in primary hemostasis VWF is tethered to subendothelial components such as various types of fibrillary collagen to mediate platelet binding and aggregation at sites of vascular injury (1), 2) at high shear rates multimeric VWF supports platelet aggregation, a function that is more effectively accomplished by larger than smaller multimers (2), and 3) in secondary hemostasis when the blood clot is formed VWF binds and stabilizes procoagulant FVIII (3–5).

Investigations on biological and biochemical functions of VWF are hampered by the fact that VWF isolated from human blood is no longer in its original form as synthesized in the endoplasmic reticulum, assembled in the Golgi and present in the blood stream immediately after secretion from Weibel-Palade bodies. Shortly after secretion, VWF is subject to proteolytic degradation by proteases. Among these proteases highly selective and specific cleavage by ADAMTS13 plays the most critical role in the regulation of the size of VWF. Thus, VWF from plasma (pdVWF) lacks high and ultra-large multimers (ULM) that are considered to be hemostatically most active. In contrast, recombinant VWF (rVWF) expressed in CHO cells is secreted in its most native form having the full spectrum of multimers present. The availability of such a VWF preparation therefore enables the study of the structure and function of VWF with all ULM present. Here we present recent studies, review existing data and put them into context with recently published biochemical studies and findings in *in vivo* models as well as in clinical studies of von Willebrand disease (VWD) patients that focus on the role of ULM.

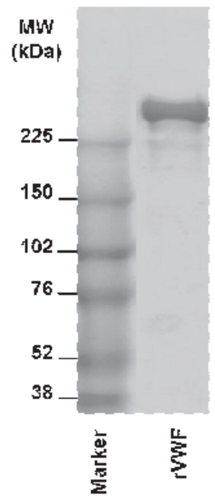

**Fig. 1** Electrophoretic analysis of the human recombinant VWF preparation in its monomeric form: SDS-PAGE (3–8 % Tris-acetate gels) – reducing conditions; Coomassie blue staining. The gel shows the purity of the rVWF preparation with a major single band with a molecular weight of approximately 270 kDa. Three faint bands appear at molecular sizes of approximately 260, 225 and 190 kDa which are also rVWF protein as these stain with a polyclonal antibody in the corresponding Western blot (not shown).

## Recombinant von Willebrand Factor rVWF (tradename in USA: VONVENDI; tradename in EU: VEYVONDI) is a highly purified recombinant human VWF produced in CHO cells by co-expression with recombinant FVIII. rVWF was developed as a recombinant therapy for treating VWD. rVWF is co-expressed with the rFVIII in CHO cells used to produce the rFVIII product, ADVATE. This rVWF may be used in conjunction with rFVIII, or as stand-alone drug product, depending on the clinical requirements. CHO cells produce partially processed and partially unprocessed rVWF still containing the pro-peptide. In order to make a consistent preparation, rVWF is exposed to recombinant furin to remove the pro-peptide. Both recombinant VWF and recombinant furin

**Tab. 1** Differences of pdVWF and rVWF

| pdVWF | rVWF |
|---|---|
| Synthesized in endothelial cells and megakaryocytes (7) | Expressed in CHO cells (13) |
| Post-translational modification of propeptide removal occurs intracellularly: during passage of the protein to the Golgi and post-Golgi compartments (7, 8) | Propeptide removal mediated *in vitro* through exposure of the pro-VWF to recombinant Furin (13) |
| Consists of VWF subunits that have been exposed to plasma ADAMTS13 (7, 10)<br>• Ultra-large VWF multimers absent<br>• Subunits cleaved at Tyr$^{1605}$-Met$^{1606}$ | Not exposed to ADAMTS13 during cell culture, purification and final container manufacturing (13)<br>• Intact VWF subunits<br>• Full spectrum of ultra-large multimers present<br>• Subunit cleavage occurs upon ADAMTS13 exposure<br>• Higher specific activity than pdVWF (13) |
| Glycosylation / ABO blood group glycans present (7, 9) | Fully glycosylated / ABO blood group glycans absent (13) |
| Plasma-derived VWF concentrates contain other proteins including ADAMTS13, hemagglutinins (7, 11, 12)<br>Recently reported adventitious agent transmission for plasma-derived VIII/VWF products<br>• Several B19V transmissions, with B19V even found in the product (14)<br>• vCJD transmission, although asymptomatic (15) | No animal or human plasma proteins added during manufacturing process<br>• No plasma protein impurities<br>• No protein used in formulation as stabilizer or bulking agent<br>• Virtually eliminates the risk of transmission of adventitious agents |

are produced under serum- and protein-free conditions. rVWF is highly purified by chromatography to a homogeneous protein preparation. For pharmaceutical use it is formulated in a protein-free buffer. ▶Figure 1 depicts the pattern of rVWF by SDS PAGE gel electrophoresis and Coomassie blue staining as present in the final drug product in its fully reduced, monomeric form (6).

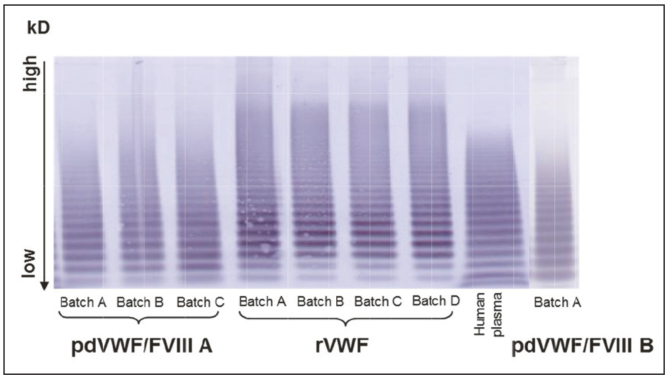

**Fig. 2** Comparison of multimer patterns of rVWF and pdVWF concentrates by low resolution (1 %) agarose gel electrophoresis; high and ULM multimers are only present in human plasma and rVWF but not in pdVWF concentrates.

## Biochemical Studies rVWF shows similarities and differences with respect to VWF derived from human plasma. ▶Table 1 compares pdVWF and rVWF (7–15). The major difference between rVWF and pdVWF is that rVWF has an intact multimer distribution. VWF stored in alpha granules of platelets and endothelial cell Weibel-Palade bodies consists of the full spectrum of multimers (8). Thus, freshly secreted VWF is composed of all multimeric forms including ULM, and is less degraded as evidenced by a reduced appearance of satellite (triplet) bands on SDS agarose gels. Only after coming into contact with proteolytic enzymes – of which ADAMTS13 is the most important – VWF is partially degraded and loses its ULM. ▶Figure 2 shows the differences in the multimeric patterns of rVWF and different pdVWF preparations. Multimer analysis demonstrates the presence of high molecular weight multimers and ULM in rVWF, which are missing in pdVWF concentrates. In contrast to pdVWF, rVWF demonstrates intact multimer bands without satellite (triplet) structure formation due to the absence of exposure to ADAMTS13 in the cell culture and during the manufacturing process (6; not shown here). Carbohydrate analysis of rVWF demonstrated an intact glycosylation pattern with a slightly higher proportion of sialylated tri- and tetra-antennary oligosaccharides than pdVWF (13). ABO antigen structures were not found on rVWF, similar to the glycosylation pattern of platelet-derived VWF. The VWF:RCo activity for rVWF is at the theoretical maximum specific activity of 1.0 IU/IU VWF:Ag and has a specific activity (VWF:RCo/protein) between 10 and 20 times higher than pdVWF. rVWF also binds effectively to collagen and exhibits higher platelet binding than pdVWF. Unexpectedly it was observed that binding of FVIII to VWF is also influenced by the multimer size (6). rVWF fractions with lower molecular weight multimers and rVWF dimers, the smallest possible unit, retained only 20% FVIII binding capacity relative to normal human plasma. These results were now confirmed in a study where a larger panel of rVWF fractions covering a broad range of multimeric sizes were investigated for their FVIII binding capacity and primary hemostatic activity determined by evaluating the ratio VWF:RCo/VWF:Ag. rVWF fractions were generated by size-exclusion chromatography as described (6) and are shown in ▶Figure 3A. FVIII binding capacity was determined by a sandwich ELISA using a commercial polyclonal rabbit antibody against human VWF (16) combined with a chromogenic assay as also previously described (6) and was calculated from a fitted reference curve constructed from a normal human plasma assumed to have 1 U of VWF:FVIIIB capacity/1 IU VWF:Ag. The results showed a gradual de-

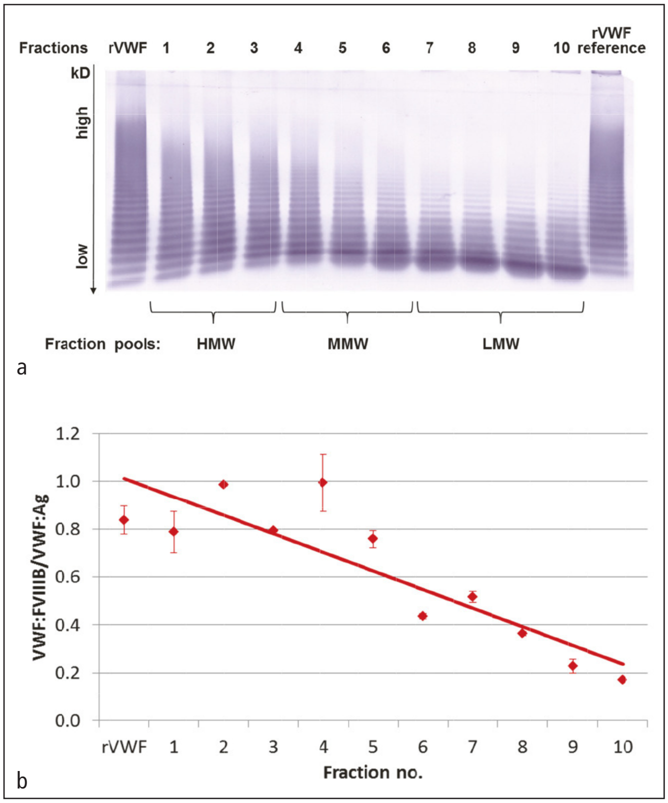

**Fig. 3** **A:** Low resolution (1 %) agarose gel electrophoresis of rVWF and fractions thereof of varying multimer size obtained by separation by size exclusion chromatography; **B:** FVIII binding measured relative to VWF:Ag content of the fractions shown in A (trendline: $R^2 = 0{,}768$); some measurements were only performed once, where error bars appear these represent means of 3 measurements ± SEM.

**Tab. 2** Characterization of rVWF fractions of varying multimer size; results of measurement of specific activity determined as VWF:Ag IU/mg protein and VWF:RCo/VWF:Ag, and FVIII binding measured relative to total protein and VWF:Ag

| Fractions | rVWF | 1 | 2 | 3 | 4 | 5 | 6 | 7 | 8 | 9 | 10 |
|---|---|---|---|---|---|---|---|---|---|---|---|
| VWF:Ag IU/mg protein | 107.3 | 127.7 | 118.8 | 128.1 | 101.9 | 138.9 | 148.0 | 133.7 | 113.3 | 136.8 | 168.2 |
| Specific activity [VWF:RCo/VWF:Ag] | 1.3 | 0.6 | 0.5 | 0.3 | 0.3 | 0.1 | 0.0 | 0.0 | 0.0 | 0.0 | 0.0 |
| VWF:FVIIIB (relative to protein content) | 90 | 100 | 117 | 101 | 101 | 105 | 65 | 69 | 41 | 31 | 29 |
| VWF:FVIIIB / VWF:Ag | 0.83 | 0.78 | 0.99 | 0.79 | 0.99 | 0.76 | 0.44 | 0.52 | 0.36 | 0.23 | 0.17 |

crease in FVIII binding towards rVWF fractions of lower multimer size (▶Table 2, ▶Figure 3B) and, thus, confirmed the correlation between rVWF multimeric size and FVIII binding capacity. Relative to the VWF:Ag content FVIII binding also decreased. As expected, the specific VWF:RCo activity was clearly dependent on the multimeric size of rVWF (▶Table 2).

## Atomic Force Microscopy Studies

Tapping mode atomic force microscopy (AFM) is a methodology enabling visualization of large molecules even of soft character like VWF on a single molecule level. AFM is unmatched in terms of high-resolution imaging under true ambient conditions that permit monitoring VWF-FVIII molecular interactions and the visualization of the proteolytic cleavage process of VWF by ADAMTS13 (17). A recently developed method could even be utilized to visualize conformational changes of rVWF and its shear dependent protein-protein interactions on the macro-molecular level (18). In a study in which rVWF was compared with a pdVWF preparation, individual molecules with a broad size distribution in terms of covalently linked monomeric units were distinguishable in pdVWF, reflecting the heterogeneous character of this protein (19). Large multimeric filaments were clearly visible, although molecules with a smaller multimer size prevailed. The larger multimers appeared to be more prominent in the AFM images of rVWF. Nevertheless, the overall structure of repeating intra-molecular globular and stretched domains resembled that of pdVWF, indicating that rVWF has the same substructural properties and assembles in the same way as pdVWF. At higher resolution AFM images showed the substructure of the molecules with sequential globular and stretched domains. rVWF was observed to undergo dramatic conformational changes upon exposure to high shear stress (shear rates up to of 31,000 $s^{-1}$) as had been reported also for pdVWF (20). Fully extended rVWF molecules in the range up to 2,300 to 2,800 nm length could be observed. This conformational change is likely to play an important role in prohemostatic VWF activation and its regulation. Stretching of VWF to a certain extent is a prerequisite for enzymatic cleavage of VWF by ADAMTS 13 (20) and degradation of VWF by ADAMTS13 depends both on time and concentration of the enzyme. *In vitro* studies demonstrated that specific degradation of VWF occurs within a few minutes after the substrate VWF had come in contact with its enzyme ADAMTS13. Human clinical studies showed that ULM disappeared within approximately three hours after the infusion of therapeutic doses of rVWF (21). ▶Figure 4 depicts the process of time-dependent proteolysis of partly stretched rVWF by exposure to ADAMTS13 demonstrated by enzymatic degradation of the large extended VWF chain (white arrows showing some cleavage points in the bottom image). This result confirmed previous findings that CHO cell derived rVWF is a good substrate for human ADAMTS 13 (22). Biomechanically this ensures that VWF, which works like a glue between platelets as well as between platelets and surface exposed structures of the subendothelium, in its stretched form only exists temporarily. The latter situation is found at the time of a vascular lesion and persists until coagulation forms the fibrin network which stabilizes finally the hemostatic plug. Almost im-

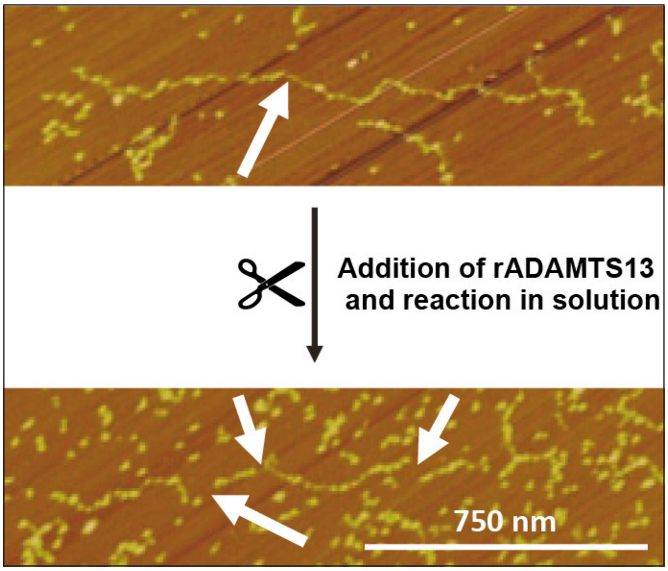

**Fig. 4** AFM (tapping mode) image of partly mechanically streched rVWF molecule (multimer – white arrow, top) and of the identical rVWF after 60 s of incubation with rADAMTS 13 (in solution) on the AFM mica target surface (white arrows are cleaving points on the rVWF, bottom).

mediately after VWF exhibits its hemostatic function it starts to be specifically proteolytically degraded and therefore downsized so that an over-exaggerated effect is prohibited.

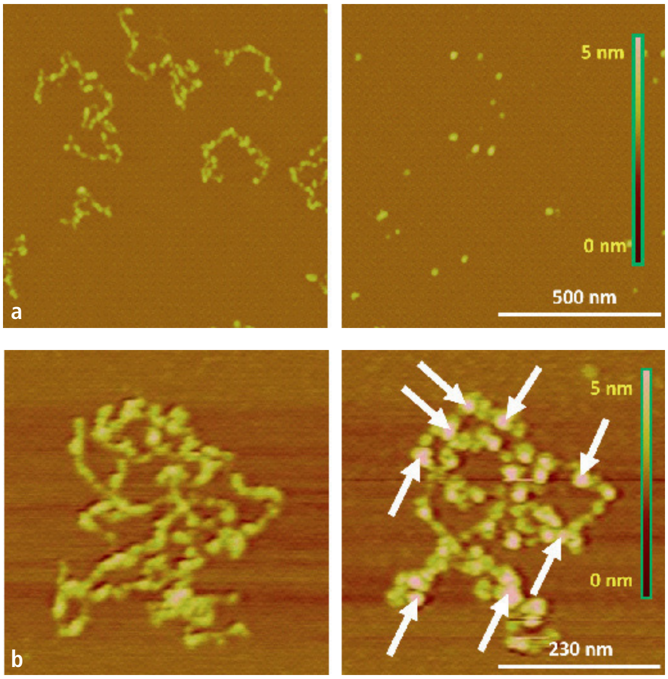

**Fig. 5** AFM (tapping mode) images of; **A:** rVWF multimer (left side) and rFVIII (right side); **B:** AFM (tapping mode) image of individual rVWF molecule (multimer, localized by means of a so-called nanoscratch) before (left side) and after (right side) incubation with rFVIII molecules (the bright light-yellowish spots indicate the attached rFVIII molecules, some are marked by white arrows).

Another AFM study suggested that rVWF can also bind rFVIII very effectively (23). First we show in ▶Figure 5A the AFM image of an individual multimeric plain rVWF molecule (left) as well as of rFVIII molecule (right) at the same magnification to provide an estimate of the size ranges related to these species. rVWF was then visualized before and after exposure to full-length recombinant FVIII (ADVATE, Shire). The AFM images in ▶Figure 5B show a single VWF molecule before and after binding with rFVIII (it is the identical molecule due to the nanoscratch approach (17) that allows its identification after the interaction in solution. A substantial number of FVIII molecules can be seen attached on a single coiled chain of VWF (right image with the white arrows marking some FVIII molecules attached on the multimeric VWF molecule, i.e. the height seen in the image is significant increased). In contrast, fully stretched rVWF does not bind FVIII, while partially stretched VWF can still bind some but less than its coiled form (17, 19). We may therefore speculate that coiled VWF, which is likely the predominant form in which VWF circulates in blood, serves as a molecular transport or delivery system that moves FVIII selectively to anatomic sites where hemostasis is required. While VWF exerting its function as "molecular glue" between platelets and components of the extracellular matrix, such as collagen, it at the same time may release non-covalently bound FVIII.

## Platelet Adhesion Studies

Platelet binding is one of the key functions of VWF. Under high shear stress, platelet adhesion is completely mediated by VWF, as VWF elongates from its globular conformation, allowing its interaction with platelet receptors. The A1 domain of VWF interacts with the glycoprotein Ib receptor of platelets, and the A1 and A3 domains both bind to collagen. To investigate the contribution of VWF multimer size to the hemostatic activity of rVWF, fractions containing distinct portions of VWF multimers were generated and analyzed for their ability to mediate platelet adhesion under shear stress *in vitro*. A routine batch of rVWF was used to generate fractions of varying multimer size. For this rVWF was chromatographically separated by size exclusion chromatography as described above for the FVIII binding studies but due to the complexity of this experiment, three pools (high, medium and low molecular weight; HMW, MMW and LMW, respectively) were prepared out of the single rVWF fractions and subjected to further analysis. (▶Figure 3A). Using these preparations, platelet interaction with collagen was studied by a perfusion chamber (BioFlux 1000z System, Fluxion BioSciences, San Francisco, CA, USA) in a 48-well plate coated with fibrillar collagen type I (Horm collagen reagent, Nycomed, Linz, Austria) and perfused with human whole blood that had been spiked with VWF samples at a final concentration of 1 IU/mL VWF:Ag or with buffer only. All perfusion experiments were carried out at a wall shear rate of 1500 $s^{-1}$. The degree of platelet adhesion was expressed as percentage of coverage of the total surface. Spiking normal human blood with rVWF resulted in an increased platelet adhesion to collagen type I, which also increased with the perfusion time. ▶Figure 6 shows the results of this experiment.

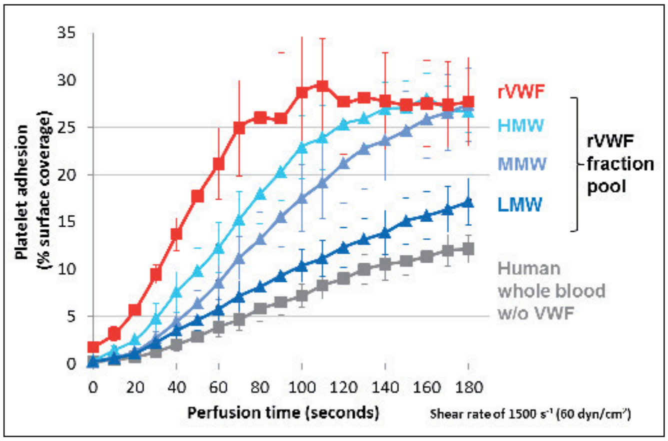

**Fig. 6** Contribution of VWF multimer size on *in vitro* platelet adhesion properties; Time-course of platelet adhesion of whole blood and blood spiked with rVWF and fractions thereof with different multimer patterns; each time point expressed as mean ±SEM, n=5

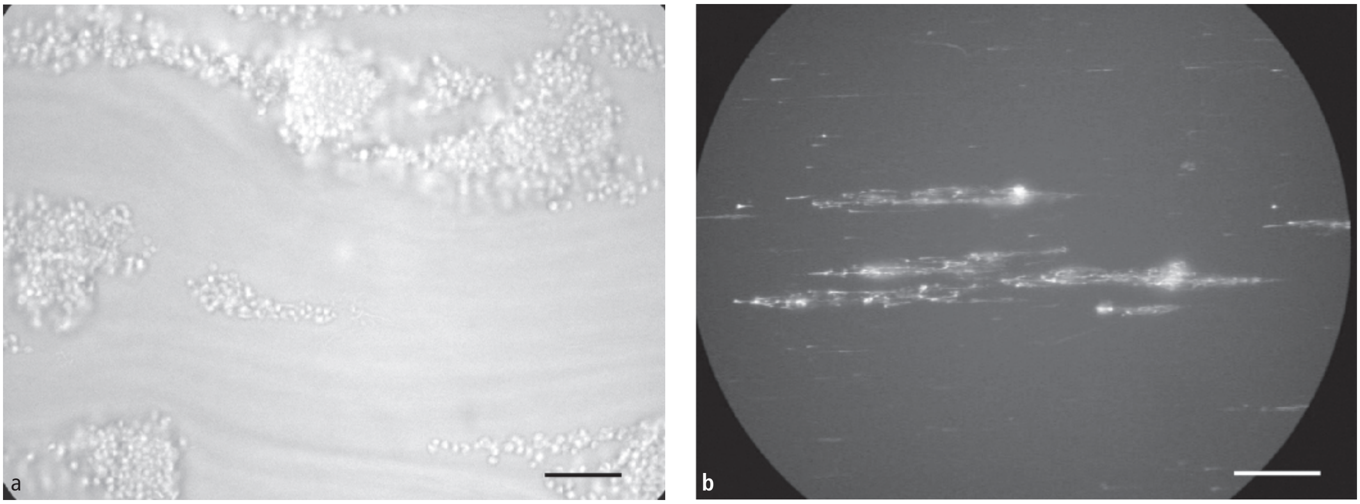

**Fig. 7** Video-microscopy still images taken at shear rate of 30,000–40,000 s-1of rolling VWF net/platelet aggregate conglomerates composed of rVWF containing ULM formed by platelets in anticoagulated whole blood rolling over a collagen type I surface. **A:** bright field (black bar represent 50 µm), **B:** fluorescence illumination (white bar represent 100 µm).

When the different rVWF fractions were used to promote platelet adhesion it was seen that the multimer size of rVWF contributed to platelet adhesion properties of VWF. Differences between rVWF and rVWF fractions "HMW" and "MMW" diminished after longer perfusion time. These results demonstrated that rVWF effectively promotes platelet adhesion to collagen under shear stress and that the platelet binding properties depend on the multimeric size of rVWF, with a higher effect observed with larger VWF multimers.

Another multimer-dependent property of rVWF was observed in a different set-up of flow experiments in which real-time visualization of platelet-VWF-interactions under high shear rates was performed (24). The experiments provided the first direct visual evidence that VWF molecules self-assemble in flow at shear rates of 20,000s$^{-1}$ and above and that the hemostatically active VWF molecules form conglomerates with platelets. The platelets were incorporated in the VWF strands network and were, at the same time, a prerequisite as well as a stabilizer for the platelet-VWF conglomerates. VWF strands and networks were seen that were up to 10µm in diameter and up to 100µm in length thus requiring VWF self-assembly in order to reach such dimensions clearly above single molecule size. These VWF structures were not only resisting the high shear stress but actually fostered by it. The experiments revealed a yet unrecognized role for ULM: formation of rolling platelet aggregates was markedly enhanced in the presence of rVWF containing ULM when compared to preparations lacking only the ULM rVWF portion but with all other multimers present. ▶Figure 7A and ▶Figure 7B show microscopic images of the formation of immobilized and rolling VWF net/platelet aggregate conglomerates on collagen type I surfaces at a shear rate of 30,000–40,000s$^{-1}$. The study suggested an improvement in efficiency of VWF binding and platelet agglomeration due to elevated shear forces which is mainly triggered by ULM. In the same set of experiments it was also shown that the effect disappeared within minutes in the presence of ADAMTS13 which highlighted the role of ADAMTS 13 to regulate VWF function.

## Animal Studies

Before human clinical studies were performed rVWF had to pass a comprehensive non-clinical study program testing safety and efficacy of rVWF in *in vitro* models and in state of the art and newly developed animal models (20, 25, 26). In animal studies focusing on the safety of rVWF, no signs of toxicity were observed in single dose toxicity studies in rats, rabbits and macaques. Substantial safety margins to the human dose could be established expressed as no observed adverse effect levels (NOAEL). In rats the NOAEL was 1400 IU VWF:RCo/kg body weight (BW) alone or co-administered with 1077 IU/kg rFVIII. In rabbits and macaques the NOAEL for rVWF was at least 1200 IU VWF:RCo/kg BW and for rVWF in combination with rFVIII (ADVATE) 600 IU VWF:RCo/kg BW with 462 IU rFVIII/kg, which were the highest doses tested in rabbits and macaques after intravenous application. *In vitro* studies demonstrated that human rVWF was a good substrate for human ADAMTS 13 and that it was rapidly cleaved by the enzyme. These studies also showed that human rVWF was only partially or slowly cleaved by ADAMTS 13 of some non-primate species (22). Tolerability of human rVWF therefore depended on how effectively large multimers were cleaved in specific animals. Nevertheless even upon very high doses of up to 1200 IU VWF:RCo/kg BW in macaques resulting in maximum levels of close to 3000 % of VWF no consumption of endogenous ADAMTS 13 was observed and also no change in platelet count were seen. These results strongly indicated that, although human rVWF contains ULM it is not hyperactive and potentially thrombogenic.

PK of rVWF compared to that of a commercially available pdVWF-FVIII preparation was evaluated in different von Willebrand deficient species. rVWF and Humate P, a pdFVIII-VWF containing concentrate were investigated in VWF-deficient dogs at the Frances OwenBlood Research laboratory, University of Chapel Hill, NC, USA. The VWF-deficient dogs have VWF levels below the limit of quantification and a lower than normal level of FVIII as a consequence of the missing VWF. Either human rVWF or Humate P was infused into two VWF-deficient dogs each at a dose of 100 IU VWF:RCo/kg. The abnormally long saline bleeding time of VWF-deficient dogs was brought to normal for a period of 4 hours after infusion of rVWF. Humate P did not correct the bleeding time. For clinical chemistry and PK determination blood samples were taken at the following time points: before and then at 5, 15, 30 min, and 1, 2, 4, 8, 12, 24, 27, 30, 48, 51, 54, 72, 78, 96, 144, 168, 192 and 216 hours after application of human VWF. Citrated plasma was prepared from blood by centrifugation at 3000 g and was stored at ≤-20 °C before analysis. All clinical chemistry variables were within the range of normal dogs, before application and 24 hours after infusion of both test items. There were no relevant changes seen in blood pressure and heart rate after infusion of rVWF or Humate P. Platelet counts were always within the normal range but a 35 % drop was observed within 15 minutes after infusion of rVWF which subsequently returned to the previous levels. Neutralizing anti-FVIII antibodies at very low levels were found 28 days after application for rVWF and Humate P whereas noticeable neutralizing anti-VWF inhibitors directed to VWF:RCo appeared at the same time. Formation of antibodies was expected because both human FVIII and human VWF are foreign proteins to the dog's immune system. VWF:Ag was determined with an ELISA using a polyclonal anti-VWF antibody (25). The VWF:Ag concentration in the samples was calculated relative to a human plasma reference preparation. FVIII activity was determined by a standard chromogenic assay. The FVIII concentration in the samples was calculated relative to the WHO plasma reference standard. The results were expressed in FVIII IU/ml. The terminal half-life, dose-adjusted area under the curve (AUC) and mean residence time (MRT) for VWF antigen and VWF:RCo activity were higher after infusion of rVWF than after Humate P injection, which is in good agreement with previous data (25). Infusion of another rVWF product into a VWF-deficient dog had resulted in a continuous increase in endogenous FVIII up to 1.6-fold over baseline level. ▶Figure 8A shows the plasma concentrations in VWD dogs relative to the maximum level found after i.v. injection of 100 IU VWF:RCo/kg BW of either rVWF or a plasma derived VWF containing FVIII preparation. The longer persistence of rVWF compared to pdVWF is apparent. In this experiment initial and terminal half-life of rVWF was calculated at 10.2 and 13.0 hours respectively, while pdVWF half-lives could be determined with 2.9 and 8.7 hours. ▶Table 3 shows PK parameters for VWF:Ag determined in VWF-deficient dogs receiving rVWF or Humate P. rVWF stabilized endogenous FVIII in VWF-deficient dogs as seen by a secondary rise in canine FVIII. This stabilizing effect of FVIII was more pronounced and lasted longer in the dog treated with rVWF then in the dog receiving pdVWF (▶Figure 8B). A similar phenomenon had been observed before in VWD mice (13).

**Tab. 3** Pharmacokinetic parameters for VWF:Ag determined in VWF-deficient dogs receiving either rVWF or a pdVWF-FVIII preparation (Humate P ) (AUC: area under the curve; MRT: mean residence time)

| Test item | *In vivo* recovery | Dose-adjusted AUC ($t_0$-$t_{inf}$) | Initial half-life | Terminal half-life | MRT | Clearance |
|---|---|---|---|---|---|---|
| | % | [(U·h/ml)/(U/kg)] | h | h | h | ml/kg |
| rVWF | 113 | 0.279 | 10.2 | 13.0 | 15.8 | 3.6 |
| Humate P | 83 | 0.102 | 2.9 | 8.7 | 9.6 | 9.8 |

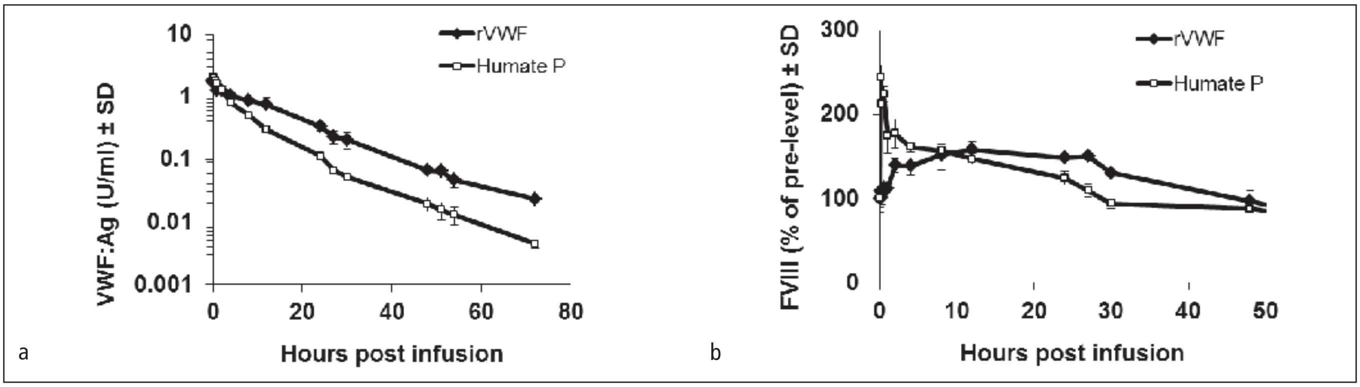

**Fig. 8** Pharmacokinetics of rVWF and a pdFVIII-VWF concentrate in von Willebrand-Deficient Dogs; **A:** rVWF has a longer half-life than pdVWF in canine VWD measured by VWF:Ag ELISA (human plasma reference); **B:** VWD dogs have reduced FVIII levels compared to normal dogs when measured by FVIII chromogenic assay (human plasma reference); human rVWF seems to stabilize canine FVIII and induces the secondary rise in FVIII levels; when normalized to starting levels FVIII remains elevated by approx. 30 % between 20 and 30 hrs after treatment with rVWF and substantially higher than after treatment with a pd FVIII-VWF concentrate (arrows).

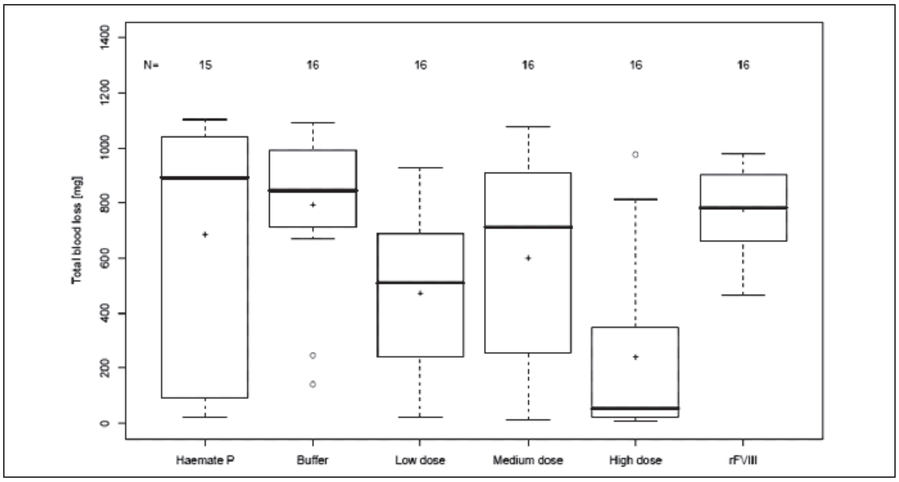

**Fig. 9** Efficacy studies with rVWF with a tail cut prophylaxis model in VWF deficient mice: Mice were treated with a pdFVIII-VWF concentrate, the equivalent amount of rFVIII and with three different doses (low dose: 400, medium dose: 600, high dose: 800 IU RCo:VWF/kg BW) of a combination of rVWF and a similar amount of rFVIII as contained based on activity determination in the pdFVIII-VWF concentrate (control: formulation buffer). Only rVWF combined with rFVIII results in cessation of bleeding demonstrating hemostatic efficacy. No bleed control seen with the pdFVIII-VWF concentrate. Box plots: cross: mean; horizontal line: median.

Humate P induced an immediate increase in FVIII because of the FVIII in the product, which was eliminated subsequently. Most likely the endogenous FVIII levels also rose at the same time.

Efficacy of rVWF in combination with rFVIII (ADVATE) in comparison to Haemate P was tested in VWF-deficient mice. Mice deficient of VWF on C57BL/6 background (27) (n = 16: 8 female and 8 male per group) were used to assess blood loss and survival in a tail-tip bleeding model. Mice were treated with either the human pdFVIII-VWF concentrate, or the equivalent doses of a combination of rVWF with human rFVIII, measured by VWF:RCo activity and FVIII one-stage clotting assays. Three different doses of rVWF (low dose: 400, medium dose: 600, high dose: 800 IU RCo:VWF/kg BW) with or without rFVIII (308, 462 and 615 IU/kg BW) and Haemate P were tested at a dose of 800 IU RCo:VWF/kg equaling the co-administration of 748 IU FVIII/kg. As controls, groups of mice were treated with human rFVIII, or the vehicle (buffer). The test and reference items were administered prophylactically 5 minutes before the tail clip. Human rVWF alone showed no efficacy at any dose in the tail-tip bleeding model in VWF deficient mice. There was no difference between mice treated with high-dose rVWF alone and the corresponding buffer. In contrast human rVWF co-injected with human rFVIII showed reduced blood loss in a linear dose-dependent manner. Prophylactic treatment with high-dose of rVWF+rFVIII led to a clearly reduced mean blood loss compared with buffer-treated animals (239 mg mean blood loss respectively 792 mg). Also only one out of 16 animals died within the observation period of 24 h whereas 9 out of 16 died in the buffer-treated group. Animals treated with Haemate P showed a mean blood loss of 682 mg, which was statistically significantly higher than in the high-dose group treated with rVWF and rFVIII. Unlike rVWF alone and pdVWF, rVWF combined with rFVIII was efficient in this mouse model (▶Figure 9). It could be speculated that the different multimeric structures between pdVWF and rVWF might be responsible for this because rVWF had an intact non-cleaved structure.

## Discussion & Conclusion rVWF completed a clinical study program consisting of a phase 1, safety, tolerability, and PK study, and a phase 3a study in which treatment of bleeding events was investigated enabling a Biologics License Application (BLA) in the USA and Marketing Authorisation Application (MAA) in Europe. In the phase 1 study safety and PK of rVWF combined at a fixed ratio with recombinant factor VIII (rFVIII) were investigated in 32 subjects with type 3 or severe type 1 von Willebrand disease (VWD) in a prospective, multicenter, randomized clinical trial. rVWF was well tolerated and no thrombotic events, inhibitors, or serious adverse events were observed. *In vivo* cleavage of ultra-large molecular-weight rVWF multimers by ADAMTS13 and generation of characteristic satellite bands was demonstrated, thus confirming the findings from preclinical studies in different animal species (25).

PK studies of rVWF alone and combined with human rFVIII (rVWF+rFVIII) in comparison to a pdFVIII/VWF were conducted in several different VWF-deficient species like mice, dogs, and pigs. Both in VWF-deficient mice and dogs the terminal half life of rVWF was longer than that of pdVWF (13, 28, 29). Statistical significance of the difference between the test items could not be addressed as only a small number of animals was investigated per test item. Despite this limited data set the results obtained in animals reflect the observations in the human clinical studies. Stabilization of endogenous FVIII in VWF deficient patients was enhanced following post-rVWF-rFVIII infusion as shown by the difference in area under the plasma concentration curve compared with pdVWF-pdFVIII. These results also confirm findings from in non-clinical *in vitro* and *in vivo* studies of improved FVIII stabilization by rVWF compared to pdVWF. The apparent difference in the stabilization of FVIII may be due to the presence of ULM in the rVWF product, the increased proportion of high molecular weight forms of multimers in the rVWF product or overall higher quality of VWF, namely non-cleaved multimers as compared to pdVWF concentrates. The observed secondary rise in FVIII suggests that rVWF administered alone as opposed to administration of the rVWF:rFVIII complex can maintain sufficient VWF and FVIII activity to treat a bleeding episode once the initial FVIII level has reached a therapeutic threshold. This was also demonstrated in a phase 3 clinical trial which evaluated the safety and hemostatic efficacy of rVWF for treatment of bleeds in severe VWD. rVWF was initially administered together with recombinant factor VIII (rFVIII) and subsequently alone, as long as hemostatic FVIII:C levels were maintained. Bleed control for all treated bleeds (N = 192 bleeds in 22 subjects) was rated excellent in 96.9 %. A single infusion was effective in 81.8 % of bleeds. FVIII:C levels increased rapidly after rVWF alone, with hemostatic levels achieved within 6 hours and sustained through 72 hours after infusion. The finding of high efficacy may be a reflection of the ULM content and longer terminal half-life of VWF:RCo of rVWF compared with pdFVIII/VWF concentrates.

Efficacy is difficult to study for VWF preparations in animals because typical bleeding models as used for determining efficacy of coagulation factors do not work. Very high doses of human VWF are required to control bleeding in VWD animals. Reduced bleeding time in VWD dogs could be demonstrated after administration of rVWF. No significant effect was observed in VWF-deficient pigs, however, indicating that the effect of rVWF appears to be influenced by species-specific considerations (23). Also, VWF deficient animals seem to require substantially higher doses of human VWF to correct their bleeding defect, which might result from different species dependent binding properties of heterologous collagen or platelet with human VWF. As a result, animal models of efficacy may not be relevant for predicting either efficacy or lack of efficacy for the products studied. While a cross-over efficacy study comparing rVWF with pdFVIII-VWF concentrates had not been performed the results from the study in VWF deficient mice presented here where mice were treated with either a human pdFVIII-VWF concentrate, or the equivalent doses of a combination of rVWF with human rFVIII shows superiority of rVWF in combination with human rFVIII to control severe bleeding. The lack of substantial observations in mice administered pdFVIII-VWF is probably due to a different composition of VWF multimers. Plasma-derived VWF consists of subunits that are cleaved at Tyr1605-Met1606 in the A2 domain of VWF. Therefore, the absence of ultra-large molecular weight forms, the fact that the VWF subunit present in pdFVIII-VWF is cleaved by ADAMTS13, and the fact that pdFVIII-VWF contains a variety of contaminating plasma proteins, including ADAMTS13, as well as citrate in the final container might influence results. rVWF is most probably more effective in the mouse model because rVWF consists of intact non-cleaved VWF subunits and also ULM which are missing in plasma derived VWF preparations. During manufacturing and down-stream processing rVWF neither comes in contact with ADAMTS13-specific proteolysis nor with any unspecific proteolytic enzyme that would cleave the protein and thus change its molecular size or reduce the contents of ULM and therefore retains the full spectrum of high molecular weight and ULM VWF missing in any VWF concentrate derived from human plasma.

*In vivo* animal data suggest that ULM are more effective in mediating primary hemostasis as also demonstrated by the pharmacological effect of DDAVP. Ultra-large molecular weight multimers are physiological in humans but are found in the circulation only under special circumstances, such as stimulation of endothelial cells after DDAVP administration. Ultra-large molecular weight multimers are also contained in platelets and are released upon platelet stimulation. DDAVP can therefore be used to treat mild hemophilia A and VWD. Upon stimulation of endothelial cells, the stored VWF is released from Weibel-Palade bodies into the circulation in the form of ULM. However, these ultra-large VWF multimers disappear within 2 hours, accompanied by an increased VWF subunit proteolysis mediated by the action of human ADAMTS13.

In contrast to pdVWF rVWF is a homogenous protein with an intact multimer pattern which it retains because it is never coming into contact with proteases that are able to degrade it, neither in the cell culture medium during the upstream process nor during downstream purification. In particular, rVWF is never exposed to ADAMTS13 which is the enzyme that by limited proteolysis generates the characteristic degradation products seen as satellite bands in agarose electrophoresis (30). This makes rVWF similar to endothelial or platelet-derived human VWF. rVWF is a highly purified recombinant protein and as such has a specific activity that exceeds any currently available plasma derived containing concentrate. The most prominent biochemical difference between pdVWF and rVWF is the presence of ULM in rVWF. These ULM explain the superior function of rVWF to promote platelet adhesion. The AFM studies suggest that a single rVWF molecule can be loaded with more than one molecule of rFVIII. This confirms previous studies which showed that at saturation, the stoichiometry was one factor VIII molecule per 50 VWF monomers, but in gel-filtration experiments VWF could be saturated by 23 times more factor VIII (31). Each mature subunit of VWF monomer contains one binding site for FVIII in the D‘-D3 domain (32) and should therefore be able to bind one FVIII molecule, provided that there is no steric hindrance. The dependency of FVIII binding capacity on the multimerization degree of rVWF was analyzed by an ELISA-type assay and surprisingly showed that the ULM portion of multimers in rVWF is also able to carry more FVIII than an equivalent amount of VWF of lower molecular size. The results described in this study confirm the previously published finding obtained with differently sized multimer fractions of a similar rVWF product (6) but are contradicting results published before with a different rVWF preparation (33). The latter study used rVWF form CHO cells which produce partially processed and partially un-processed rVWF still containing the pro-peptide and therefore an uncontrolled amount of pro-VWF which is unable to bind FVIII. This is in contrast to our rVWF preparation which is treated with recombinant furin to remove the pro-peptide and to make it consistent in its ability to bind FVIII. This difference in the rVWF preparations likely explains the different outcomes and interpretation of the FVIII binding studies. If FVIII binding would be strongly dependent on multimer size, then one would expect FVIII/VWF:Ag ratios to be reduced in patients with VWF-type 2A and increased in patients with TTP resulting from ADAMTS13 deficiency. However, current textbook knowledge describes FVIII levels of VWD-type 2A patients as low or normal and a recent larger study found FVIII:VWF:Ag ratios quite normal in VWD-type 2A (34). Other literature described how heterogeneous patients with VWD-type 2A could be in their FVIII levels (35). Similarly FVIII levels are not substantially increased in TTP which is not always easy to determine, given the inflammatory nature that is often associated with this disorder. Therefore at this point in time it remains open if a sustained increase in ULM concomitantly would increase FVIII levels. Another possible explanation for the increased FVIII-binding capacity of rVWF compared to pdVWF in the studies discussed in this manuscript is the non-exposure of rVWF to ADAMTS13. It has been previously described that high-affinity FVIII binding sites are located on N-terminal disulfide-linked VWF subunits (36). Unlike VWF in plasma that has been exposed to ADAMTS13 resulting in the formation of differently-sized multimer combinations, multimers of rVWF are of homogenous structure. As a consequence of these structural differences, the relative number of N-terminal linkages of a lower-sized VWF in plasma is higher than of rVWF fraction of comparable multimeric size and protein mass, thus explaining the lower FVIII binding capacity of rVWF fractions of lower multimer size.

The finding with the fully matured rVWF product indicates a functional role of ULM that goes beyond the primary hemostatic function of VWF to work as the glue between platelets and sub-endothelial structures necessary to form a hemostatic plug. Interaction between FVIII and VWF can be measured by many different technologies but AFM for the first time allows visualization of the interaction. Recently published studies performed by AFM indicate that changes in the superstructure of VWF dependent on stretching induced by shear force can change its ability to bind FVIII (37).These studies suggest that stretched VWF releases FVIII which would be in line with the current knowledge that fully stretched VWF binds to platelets and at the same time releases FVIII to function as a cofactor in the tenase complex, where the enzyme (FIXa)-substrate (FX) reaction catalyzed by FVIII occurs to generate FXa. It remains subject of further research to which extent such a mechanism triggered by unfolding of VWF adds to release of FVIII from VWF occurring through activation of FVIII by local thrombin. ULM VWF seems to be capable of doing more than that. Another set of experiments gave the first direct visual evidence that rVWF molecules reversibly self-assemble in flow at high shear rates and form conglomerates with platelets underlining it superior gluing effect. Future studies with complementary *in vitro* and *in vivo* models will be required to confirm this superior effect of rVWF when comparing to VWF obtained from human plasma. In parallel, upon clinical use of rVWF, it will be demonstrated in humans if the superior effect found in animals and in the biochemical studies would also translate into improved treatment outcomes in patients with VWD.

## Conflict of interest

P.L. Turecek, M. Turecek, H. Gritsch, O. Obermann-Slupetzky, and G. Schrenk are full-time employees of Baxalta Innovations GmbH, Vienna, Austria
M. Spannagl, and T. Kragh performed the work as investigator initiated research without funding and have therefore no conflict of interest to report.
G. Allmaier initiated and supervised the work as part of a project funded by Baxalta Innovations GmbH, Vienna, Austria
B.M. Ewenstein is a full-time employee of Shire, Cambridge, MA, USA
L. Valentino is a full-time employee of Shire, Chicago, IL, USA

## Authorship contribution

P.L. Turecek conceived the work described in the manuscript and wrote the manuscript
M. Spannagl and T. Kragh performed parts of the platelet adhesion studies as presented by the video-microscopy images
G. Allmaier initiated and summarized the tapping mode atomic force microscopy studies
M. Turecek described the animal studies
H. Gritsch performed the analysis for the animal studies
G. Schrenk performed parts of the platelet adhesion and FVIII binding studies
O. Obermann-Slupetzky and B.M. Ewenstein provided data analysis and interpreted the human clinical studies
L. Valentino reviewed the manuscript
